# Multiple Susceptibility Loci for Radiation-Induced Mammary Tumorigenesis in F2[Dahl S x R]-Intercross Rats

**DOI:** 10.1371/journal.pone.0072143

**Published:** 2013-08-14

**Authors:** Victoria L. Herrera, Lorenz R. Ponce, Nelson Ruiz-Opazo

**Affiliations:** Department of Medicine, Boston University School of Medicine, Boston, Massachusetts, United States of America; National Cancer Institute, United States of America

## Abstract

Although two major breast cancer susceptibility genes, *BRCA1* and *BRCA2,* have been identified accounting for 20% of breast cancer genetic risk, identification of other susceptibility genes accounting for 80% risk remains a challenge due to the complex, multi-factorial nature of breast cancer. Complexity derives from multiple genetic determinants, permutations of gene-environment interactions, along with presumptive low-penetrance of breast cancer predisposing genes, and genetic heterogeneity of human populations. As with other complex diseases, dissection of genetic determinants in animal models provides key insight since genetic heterogeneity and environmental factors can be experimentally controlled, thus facilitating the detection of quantitative trait loci (QTL). We therefore, performed the first genome-wide scan for loci contributing to radiation-induced mammary tumorigenesis in female F2-(Dahl S x R)-intercross rats. Tumorigenesis was measured as tumor burden index (TBI) after induction of rat mammary tumors at forty days of age via ^127^Cs-radiation. We observed a spectrum of tumor latency, size-progression, and pathology from poorly differentiated ductal adenocarcinoma to fibroadenoma, indicating major effects of gene-environment interactions. We identified two mammary tumorigenesis susceptibility quantitative trait loci (*Mts*-QTLs) with significant linkage: *Mts-1* on chromosome-9 (LOD-2.98) and *Mts-2* on chromosome-1 (LOD-2.61), as well as two *Mts*-QTLs with suggestive linkage: *Mts-3* on chromosome-5 (LOD-1.93) and *Mts-4* on chromosome-18 (LOD-1.54). Interestingly, Chr9*-Mts-1*, Chr5*-Mts-3* and Chr18*-Mts-4* QTLs are unique to irradiation-induced mammary tumorigenesis, while Chr1*-Mts-2* QTL overlaps with a mammary cancer susceptibility QTL (*Mcs* 3) reported for 7,12-dimethylbenz-[α]antracene (DMBA)-induced mammary tumorigenesis in F2[COP x Wistar-Furth]-intercross rats. Altogether, our results suggest at least three distinct susceptibility QTLs for irradiation-induced mammary tumorigenesis not detected in genetic studies of chemically-induced and hormone-induced mammary tumorigenesis. While more study is needed to identify the specific Mts-gene variants, elucidation of specific variant(s) could establish causal gene pathways involved in mammary tumorigenesis in humans, and hence novel pathways for therapy.

## Introduction

Breast cancer is one of the most prevalent female cancers in the world, affecting at least 10% of women in industrialized nations [1], [2]. Breast cancer is a complex multifactorial trait encompassing genetic and environmental factors [3], [4]. To date few breast cancer susceptibility genes have been identified in human populations with BRCA1 and BRCA2 variants accounting for less than 20% of the genetic risk of breast cancer [5]. Due to the complex inheritance of this disorder and genetic heterogeneous nature of human populations it has been difficult to unravel novel breast cancer susceptibility/resistance genes that could elucidate novel pathways for diagnosis, treatment and prevention of breast cancer.

Two classes of rat models of mammary carcinogenesis have been frequently used; chemically-induced mammary carcinogenesis using compounds like the polycyclic aromatic hydrocarbon 7,12-dimethylbenz[*a*]anthracene (DMBA) [6], *N*-nitroso-*N*-methylurea (NMU) [7], 2-amino-1-methyl-6phenylimidazo[4,5-*b*]pyridine (PhIP) [8], estrogens [9] and radiation-induced mammary carcinogenesis [10]–[15]. Of the few reported genetic studies that have been performed in animal models of mammary carcinogenesis, all have utilized the chemically-induced model as the chosen animal model system [3]. Thus, QTLs affecting susceptibility to mammary tumors have been reported in rat intercrosses subjected to DMBA-induced [16] and 17β-Estradiol-induced [17] mammary carcinogenesis. However, there are no reports on genetic studies performed on radiation-induced mammary carcinogenesis, despite the fact that ionizing radiation is one of the few well-characterized etiologic factors of human breast cancer [18] and the well-established fact that the female breast is one of the most susceptible organs to radiation-induced cancer [19]–[22].

The objective of this study was to perform a genome scan for QTLs affecting radiation-induced tumorigenesis. The sample used was an F2 (Dahl S x R)-intercross female rat population phenotyped for radiation-induced mammary tumorigenesis after induction of rat mammary gland tumors at 40 days of age via ^127^Cs-radiation. We chose the Dahl S/Dahl R rat model because these two inbred rat lines were derived from the Sprague-Dawley rat [23], one of the rat strains most susceptible to radiation-induced mammary carcinogenesis [24]–[26].

## Results

**Figure 1:**
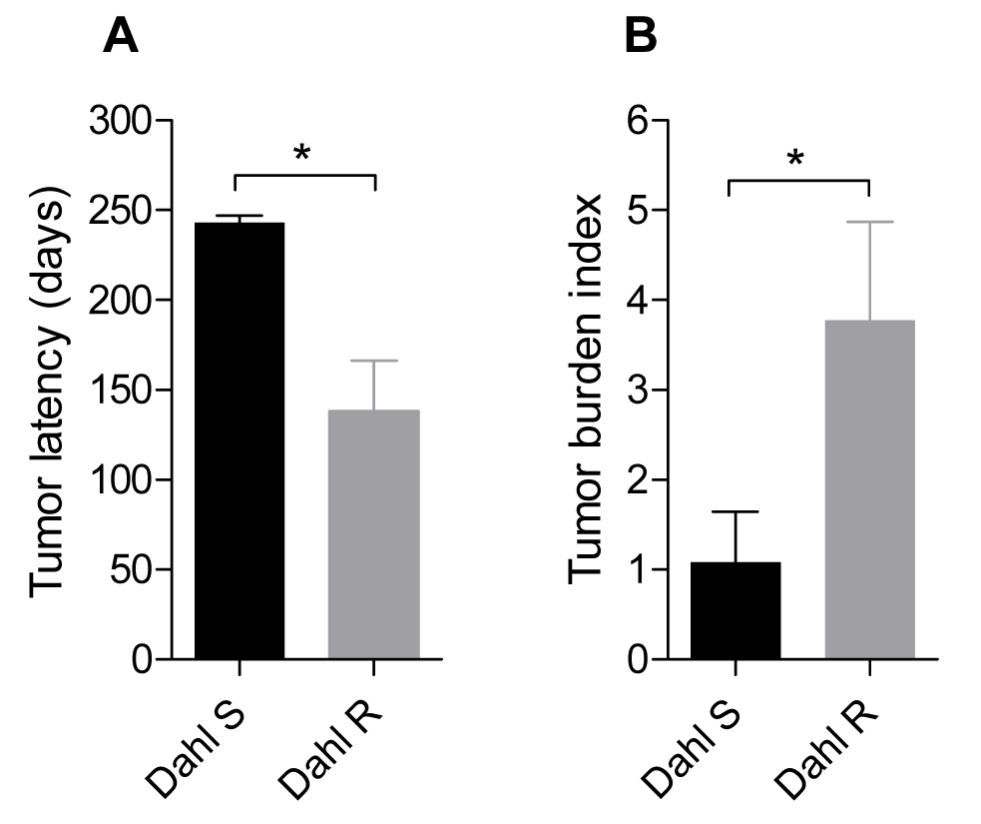
Tumor development in Dahl S and Dahl R female rats. The tumor latency (A) and tumor burden index (B) for Dahl S (black bars) and Dahl R (gray bars) female rats are presented. ^*^,*P*<0.05 unpaired *t*-test.

In order to assess potential differences in susceptibility to radiation-induced tumorigenesis we first analyzed inbred parental strains, Dahl S and Dahl R female rats, selected for the observed tendency to develop spontaneous mammary tumors in F2 [Dahl S x R]-intercross rats. We measured latency to tumor development and number of mammary gland tumors after a single dose of irradiation at 40 days of age in parental Dahl S and Dahl R female rats. As shown in Figure 1, Dahl R rats exhibited decreased tumor latency and increased tumor burden index compared with Dahl S rats (*P*<0.05), indicating increased radiation-induced breast cancer susceptibility in Dahl R female rats.

**Figure 2:**
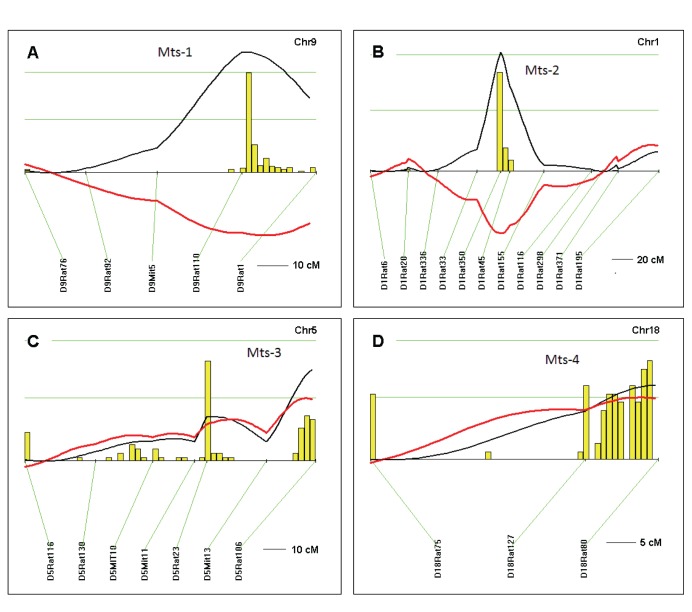
QTLs for mammary tumorigenesis susceptibility (Mts) in F2 [Dahl S x R]-intercross female rats. Chromosomes with significant and suggestive QTLs were analyzed by interval mapping with bootstrap resampling method to estimate a confidence interval (QTXb19 Map Manager): Panel A, chromosome 9 (*Mts-1*); B, chromosome 1 (*Mts-2*); C, chromosome 5 (*Mts-3*); D, chromosome 18 (*Mts-4*). Yellow histograms represent the bootstrap-based confidence intervals for the detected QTLs. Orientation of chromosomes: left → right starting from lowest Mbp. Horizontal green lines mark LOD values for significance of linkage, from top to bottom: significant LOD ≥2.48; suggestive LOD ≥1.30; LOD (black line); regression coefficient for additive effect (red line).

**Figure 3:**
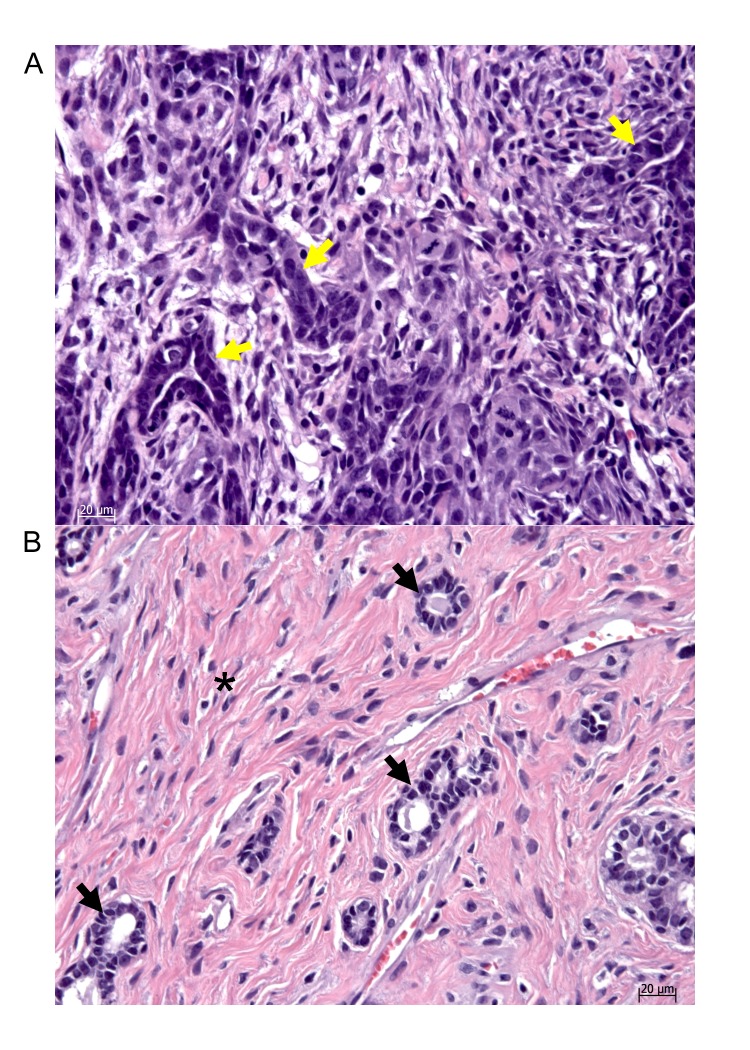
Hematoxylin and Eosin stained digital photomicrographs of representative breast tumor phenotypes in the F2-cohort of radiation-induced breast tumors. A, Adenoductal carcinoma phenotype, B, fibroadenoma. →, representative duct morphology in each section; *, hyaline (pink) fibrous tissue markedly increased in fibroadenoma.

We next performed a total genome scan for QTLs affecting radiation-induced mammary tumorigenesis susceptibility (*Mts*) using 150 F2 (Dahl S x R)-intercross female rats phenotyped for tumor burden index (TBI) as the quantitative measure for tumorigenicity measuring both latency to tumor formation and number of tumors. We detected two *Mts*-QTLs with significant linkage: (*Mts-1* on chromosome 9, LOD 2.98 and *Mts-2* on chromosome 1, LOD 2.61; Table 1 and Figure 2). We also detected two *Mts*-QTLs with suggestive linkage (*Mts-3* on chromosome 5, LOD 1.93 and *Mts-4* on chromosome 18, LOD 1.54; Table 1 and Figure 2). Additional analysis for interactive effects on breast cancer susceptibility reveals no gene-gene interaction in this F2 (Dahl S x R)-intercross female rat cohort. Histopathological analysis of representative Hematoxylin and Eosin stained sections from seven rats detected mammary adenocarcinomas in five, including poorly differentiated adenocarcinoma (Figure 3), and fibroadenomas in two (Figure 3). This is similar to observations by Cronkite et al 1960 [27], and suggest the paradigm that tumorigenesis induced by a given environmental factor, irradiation, produces a spectrum of mammary tumor pathology.

**Table 1:** QTLs for mammary tumorigenesis susceptibility in F2 (Dahl S x R)-intercross female rats.

QTL	Location	LOD	%	QTL^a^	Candidate^b^
Mts-1	Chr9∶84–104 Mbp	2.98 (**S**)	9 (↓)		Prlh
Mts-2	Chr1∶125–145 Mbp	2.61 (**S**)	8 (↓)	Mcs3[16]	Anpep orBlm
Mts-3	Chr5∶147–167 Mbp	1.93 (**Sug**)	6 (↑)		Ece1
Mts-4	Chr18∶66–86 Mbp	1.54 (**Sug**)	5 (↑)		Smad2

QTL, quantitative trait locus; Mts, mammary tumorigenesis susceptibility; Chr, chromosome; %, the amount in % of total trait variance that would be explained by a QTL at these loci; Mbp, mega-base pair; LOD, logarithm of the odds score derived from the likelihood ratio statistic using a factor of 4.6; ↑, S-allele increases trait; ↓, S-allele decreases trait. Significance determined from 2000 permutations on data set: LOD 2.48 significant (S); LOD 1.30 suggestive (Sug).

a Overlapping QTL detected in previous study: *Mcs* = mammary cancer susceptibility.

b candidate genes in rat-human syntenic regions; *Prlh*, prolactin hormone; *Anpep*, alanyl (membrane) aminopeptidase; *Blm*, Bloom syndrome homolog; *Ece1*, endothelin-converting enzyme-1; *Smad2*, SMAD family member 2.

Although QTLs still need to be narrowed to pinpoint causal genes for tumorigenicity, and specific variants identified, analysis of syntenic regions to *Mts-1 to Mts-4* in humans reveals candidate genes previously implicated in breast cancer. On chromosome 9, *Prlh* (prolactin releasing hormone) is a candidate gene for *Mts-1 *QTL since *Prlh* maps to rat chromosome-9 at coordinate 90.15 Mbp within the *Mts-1* QTL region (Table 1). Prlh modulates secretion of prolactin [28], a hormone that has been shown to be a risk factor for human breast cancer [28], [29]. Notably, for *Mts-2 *QTL on chromosome-1, the marker at the QTL-peak, D1Rat350, is located within the *Anpep* [alanyl (membrane) aminopeptidase] transcription unit, an enzyme that might be a candidate gene because it has been associated with invasive colorectal cancer [30] and prostate cancer [31], and Barrett’s adenocarcinoma [32]. Another candidate gene on chromosome 1, *Blm* localizes at 136.2 Mb also within the *Mts-2* QTL interval (Table 1). Notably, *Blm* (Bloom syndrome homolog) has been implicated in breast cancer susceptibility in humans [33], [34]. The *Mts-3* chromosome-5 QTL region (Table 1) harbors *Ece1* (endothelin-converting enzyme-1 at 156.6 Mb), an enzyme that has also been implicated in human breast cancer [35], [36]. Finally, within the *Mts-4* interval on chromosome 18 (Table 1) resides *Smad2* (SMAD family member 2/mothers against decapentaplegic homolog 2, 73.18 Mb), a signaling protein whose phosphorylation mediates TGF-beta induced breast cancer invasiveness [37], [38].

## Discussion

This is the first genome-wide scan for QTLs affecting radiation-induced mammary tumorigenesis in rodents. We detected two significant *Mts*-QTLs on chromosomes 9 and 1 and two suggestive *Mts*-QTLs on chromosomes 5 and 18. The chromosome 9 *Mts-1*, chromosome 5 -*Mts*-*3* and chromosome 18 *Mts-4* QTLs represent novel QTL regions associated with mammary tumorigenesis not previously observed in other rat intercrosses of DMBA-induced and estrogen-induced mammary tumorigenesis [3]. *Mts-2* QTL spanning chromosome1 125–145 Mbp region overlaps with *Mcs3* QTL that maps to chromosome1 109.1–138.8 Mbp region in a COP x WF intercross rat population phenotyped for DMBA-induced mammary carcinogenesis [16]. Interesting, both QTLs decrease susceptibility to tumorigenesis suggesting that the same gene might underlie both QTLs effects on mammary tumorigenesis. *Mts-3* mapping to chromosome 5 147–167 Mbp region partially overlaps with *Emca1* spanning chromosome 5 107–159 Mbp region detected in an F2 (COP x ACI)-intercross rat population characterized for estrogen-induced mammary carcinogenesis [39]. However, having different modes of inheritance, recessive for *Emca1* and co-dominant or additive for *Mts-3*, data suggest that different genes might account for these two QTL effects on rat mammary tumorigenesis. Finally, *Mts-4* at chromosome 18 66–86 Mbp region partially overlaps with *Mcsta2* that peaks at 68 Mbp in a SPRD-Cu3 x WKY backcross rat population characterized for DMBA-induced mammary carcinogenesis [40]. However, *Mts-4* and *Mcsta* have opposite effects on susceptibility with *Mcsta* decreasing and *Mts-4* increasing susceptibility to mammary tumorigenesis suggesting that different genetic determinants underlie these QTLs effects on tumorigenesis. Altogether, the data suggest that distinct genetic determinants exist that confer susceptibility to irradiation-induced mammary tumorigenesis from those loci affecting chemically-induced and estrogen-induced mammary tumorigenesis.

As described by Cronkite et al 1960 [27], radiation-induced mammary tumor models exhibits both adenocarcinoma and fibroadenoma in Sprague Dawley rats. Notably, both adenocarcinoma and fibroadenoma were also detected in the F2[Dahl S x R] breast cancer cohort studied here concordant with the fact that both Dahl S and Dahl R rats were derived from Sprague Dawley rats selected for salt-sensitive and salt-resistant hypertension respectively. Given the same environmental insult, the spectrum of pathologies from malignant to benign, and the detection of multiple QTLs suggest that susceptibility to mammary tumorigenesis is a complex multifactorial event likely involving multiple genetic determinants and genetic modifiers. As spectrum of susceptibility, the data suggest that genetic analysis for sporadic breast cancer and fibroadenoma can be analyzed as one pathogenic event with a spectrum much like other diseases.

Although further studies are needed to identify causal genes in respective *Mts-*QTLs, the panel of candidate genes with reported gene expression changes or polymorphisms in breast cancer patients, *Prlh* for *Mts-1, Anpep* or *Blm* for *Mts-2, Ece1* for *Mts-3*, and *Smad2* for *Mts-4*_validate the hypotheses that these genes should be studied further in different experimental systems and in humans as potential susceptibility genes for mammary tumorigenesis. Although no statistically significant genetic interaction was detected among the QTLs, we note all *Mts*-QTLs 1*–*4 candidate genes are associated with key aspects of breast tumor progression and malignancy, which collectively could increase tumorigenesis susceptibility. Increased *Prlh* leading to higher prolactin levels is associated with increased risk for breast cancer, increased metastasis, disease progression, lower response to tamoxifen and worse clinical prognosis [41]. Furthermore, *Prlh* as a candidate gene for *Mts-*1 QTL is concordant with the observation that prolactin accelerated mammary tumorigenesis initiated by radiation [42]. Similarly, the candidate genes for *Mts-2* QTL, *Anpep* or *Blm* are both implicated in breast cancer. Anpep is increased in breast cancer effusions [43], and its down regulation is associated with invasive colorectal cancer [30] and prostate cancer [31], while over expression of Anpep has been linked to Barrett’s adenocarcinomas [32]. *Blm*, the gene for Bloom syndrome, is a DNA repair gene which may play a role in breast cancer occurrence as its loss may contribute to somatic mutations and loss of heterozygosis, chromosomal instability, aneuploidy, and sensitivity to DNA damaging agents [44]. For *Mts-3* QTL candidate gene, *Ece1*, cumulative studies point to its role in breast cancer invasiveness and more frequent recurrence [45]. Lastly, *Mts-4 *QTL candidate gene, *Smad2,* underlies Smad2-dependent epithelial mesenchymal transition of breast cancer cells [46], a key step in invasiveness and subsequent metastasis. Intriguingly, the deduced synergisms from these breast cancer roles for each *Mts* QTL candidate gene suggest the hypothesis that QTL-burden could increase risk, reiterating the need for further study.

Further inspection of the Rat Genome Database (RGD) reveals additional candidate genes within *Mts-1*, *Mts-2*, *Mts-3* and *Mts-4* QTLs that have been associated with different types of cancers (Table 2). However, more studies involving fine mapping through sub-strain construction will be necessary to identify the genes underlying these QTLs.

**Table 2:** Genes associated with cancer within mammary tumorigenesis susceptibility QTLs detected in F2 (Dahl S x R)-intercross female rats.

Symbol	Description	Location (nt)	Association [ref]
**Mts-1** (Chr9)			
Ugt1a7c	UDP glucuronosyltransferase 1 family, polypeptide A7C	87029500–87098362	Pancreatic cancer [47]
Ugt1a1	UDP glucuronosyltransferase 1 family, polypeptide A1	87091241–87098362	Endometrial cancer [48], ovarian cancer [49]
Pam	Peptidylglycine alpha-amidating monooxygenase	96893071–97047523	Prostate cancer [50]
Ralbp1	RalA binding protein 1	104617400–104653856	Bladder cancer [51]
**Mts-2** (Chr1)			
Ntrk3	Neurotrophic tyrosine kinase, receptor, type 3	133925530–134302139	Pancreatic cancer [52]
Fzd4	Frizzled family receptor 4	145953743–145957666	Prostate cancer [53]
**Mts-3** (Chr5)			
Hdac1	Histone deacetylase 1	148672515–148699810	Breast cancer [54], cervical cancer [55],endometrial cancer [56], ovarian cancer [56]
Sfn	Stratifin	151475395–151479996	Breast cancer [57], endometrialcancer [58], prostate cancer (57)
Runx3	Runt-related transcription factor 3	153950116–153973141	Breast cancer [59], ovarian cancer [60],colorectal cancer [61]
Wnt4	Wingless-type MMTV integration site family, member 4	156064371–156083198	Endometrial cancer [62]
Sdhb	Succinate dehydrogenase complex, subunit B, iron sulfur(Ip)	159818669–159839772	Renal cancer [63]
Casp9	Caspase 9, apoptosis-related cysteine peptidase	160704234–160721796	Breast cancer [64]
Tnfrsf1b	Tumor necrosis factor receptor superfamily, member 1b	163666541–163697484	Renal cancer [65]
**Mts-4** (Chr18)			
Smad4	SMAD family member 4	70432705–70461541	Ovarian cancer [66], endometrial cancer [67]
Smad7	SMAD family member 7	72294803–72323354	Endometrial cancer [68]

Table legend: Genes and gene locations on rat chromosomes 1, 5, 9 and 18 regions as per RGD. nt, nucleotide; ref, reference.

In summary, the genome-wide scan for QTLs influencing breast cancer susceptibility identified genetic linkage to chromosomes 9, 1, 5 and 18 with radiation-induced mammary tumorigenesis in Dahl rats. The chromosomes 9, 5 and 18 QTLs were unique to this F2 (Dahl S x R)-intercross rat population suggesting that genetic mechanisms underlying radiation-induced mammary tumorigenesis differs substantially from mechanisms involved in chemically-induced and hormone-induced mammary tumorigenesis. Histopathology observations of a spectrum from poorly differentiated adenocarcinoma to benign fibroadenoma despite exposure to identical radiation dose in our F2[Dahl S x R]-intercross rat mammary tumor cohort suggest the importance of genetic susceptibility and/or gene modifiers in this gene-environment interaction paradigm.

Altogether, the detection of *Mts-* QTLs spanning fibroadenoma to poorly differentiated adenocarcinoma suggest the paradigm that genetic analysis of tumorigenesis-QTLs in humans could help elucidate breast cancer susceptibility genes which have remained elusive to date. Alternatively, the detection of candidate genes within *Mts-*QTLs associated with breast cancer in humans validates their analysis as causal susceptibility genes in human cohorts respectively. Elucidation of variants accounting for mammary tumorigenesis in Dahl rats will give insights into gene pathways important for gene-environment interactions, tumor initiation and progression.

## Materials and Methods

### Ethics Statement

This study was performed in strict accordance with the recommendations in the Guide for the Care and Use of Laboratory Animals of the National Institutes of Health. The protocol was approved by the Committee on the Ethics of Animal Experiments of Boston University School of Medicine (Permit Number: AN-13924).

### Genetic Crosses

Dahl S/jrHsd and Dahl R/jrHsd rats were obtained from Harlan (Indianapolis, Indiana). Parental strains (Dahl R/jrHsd female x Dahl S/jrHsd male) were crossed to produce F1 progeny. The F2 subjects were derived from brother-to-sister mating of F1 hybrids to produce the F2 female (n = 150) segregating population.

### Phenotypic Characterization and Genotyping

Animals were maintained on a LabDiet 5001 rodent chow (Harlan Teklad, Madison WI) containing 0.23% NaCl. We induced rat mammary gland tumors in 12 Dahl S/jrHsd, 12 Dahl R/jrHsd and 150 F2 female subjects as described [27] at 40 days of age via ^127^Cs-radiation. Rats were exposed to 400 r of whole body radiation. All subjects were sacrificed when tumor burden reached a total of 3 cm in diameter for animals harboring one or multiple tumors or at 12 months of age for those that did not develop tumors. Three out of 12 Dahl S/jrHsd, seven out 12 Dahl R/jrHsd and ninety eight out of 150 F2 female rats developed mammary tumors. Seven F2 female rat tumors were randomly selected for histological analysis (five were found to be carcinomas and two were fibroadenomas). Routine Hematoxylin and Eosin stained histological sections were obtained from 4% paraformaldehyde fixed tumor tissues and analyzed with a clinical tumor pathologist (Michael J O’Brien, MD, MPH) at Boston Medical Center. Tumor latency was computed from 40 days of age (at time of radiation). Tumor burden index was computed using the formula TBI = 1+[Tx2]+[TL_H_/TL] (T = # tumors; TL_H_ = highest tumor latency; TL = tumor latency) as described [69]. We used 112 microsatellite markers informative for our F2 (Dahl S x R)-intercross spanning the whole genome (including all 20 autosomes plus the X chromosome) with an average marker density of 12.7 cM. Microsatellite markers were PCR genotyped and detected by 6% denaturing polyacrylamide gel electrophoresis.

### Intercross Linkage Analysis

Phenotype distributions were analyzed for normality; data transformations were done when necessary and datasets that passed Kolmogorov-Smirnov normality testing (SigmaPlot11.2) were used for linkage analysis. QTL analysis was performed using the tumor burden index (TBI) as quantitative trait that was computed using the formula TBI = 1+[Tx2]+[TL_H_/TL] (T = # tumors; TL_H_ = highest tumor latency; TL = tumor latency) as described [69]. Linkage maps, marker regression and composite interval mapping were done with the Map Manager QTXb19 (MMQTXb19) program for windows [70] which generates a likelihood ratio statistic (LRS) as a measure of the significance of a possible QTL. Genetic distances were calculated using Kosambi mapping function (genetic distances are expressed in centiMorgan, cM). Critical significance values (LRS values) for interval mapping were determined by a permutation test (2000 permutations at all loci tested) on our female cohort using Kosambi mapping function and an additive regression model. Values for suggestive linkage LRS = 6.0 (LOD 1.30) and for significant linkage LRS = 11.4 (LOD 2.48). LRS 4.6 delineates LOD 1-support interval. Confidence interval for a QTL location was estimated by bootstrap resampling method wherein histogram single peak delineates the QTL and peak widths define confidence interval for the QTL. Histograms which show more than one peak warn that the position for the QTL is not well defined or that there may be multiple linked QTLs (QTX Map Manager). We also performed interaction analysis using the Map Manager QTXb19 program applying a two-stage test paradigm for determination of interaction in which the pair of loci must pass two tests in order to be reported as having a significant interaction effect. First, the significance of the total effect of the two loci must be <0.00001 and second, the pairs of loci must exhibit a *P* value <0.01 for the interaction effect.
